# Supplementary material for: Precise regulation of the guidance receptor DMA-1 by KPC-1/Furin instructs dendritic branching decisions
Source: eLife. 2016 Mar 14;5:e11008. doi: 10.7554/eLife.11008 (PMC4811766; doi:10.7554/eLife.11008)
Supplement: Supplementary file 1. — Table S2 Plasmids used in this study. Table S3 sgRNAs and repair oligos for CRISPR. DOI: http://dx.doi.org/10.7554/eLife.11008.019 [file elife-11008-supp1.docx]

**Supplemental information**

**Table S1. Mutant alleles and transgenes used in this study**

1. Mutant alleles

| **Allele** | **Reference** | **Figure** |
| --- | --- | --- |
| *kpc-1(gk8)* | ([Schroeder et al., 2013](#_ENREF_4)) | 1, 1S1, 2, 2S1, 2S2, 2S3, 4, 4S1, 4S2, 5S1, 7 |
| *kpc-1(xr58)* | This study | 3, 3S1 |
| *kpc-1(wy1060)* | This study | 3S1 |
| *sax-7(nj48)* | ([Sasakura et al., 2005](#_ENREF_3)) | 2, 2S1, 2S2, 2S3 |
| *mnr-1(wy758)* | ([Dong et al., 2013](#_ENREF_1)) | 2, 2S1, 2S2 |
| *dma-1(wy686)* | ([Liu and Shen, 2012](#_ENREF_2)) | 2, 2S1, 2S2 |
| *dma-1(wy908)* | This study | 5S1 |
| *dma-1(wy1041)* | This study | 4 |

1. Integrated transgenes

| **Name** | **Chromosome** | **Constructs** | **Co-injection marker** | **Figure** |
| --- | --- | --- | --- | --- |
| *wyIs50001* | unknown | pXD26, pOL036 | P*odr-1::rfp* | 2S1 |
| *wyIs592* | III | pOL020 | P*odr-1::rfp* | 1S1, 2, 2S2, 3, 3S1, 4S2, 5, 5S1 |
| *wyIs369* | IV | pOL071 | P*myo-2::mCherry* | 2 |
| *wyIs733* | X | pXD302 | P*odr-1::gfp* | 7 |
| *qyIs369* | X | pWZ55 | *unc-119(+)* | 4 |
| *qyIs366* | IV | pWZ54 | *unc-119(+)* | 4S1 |

1. Extrachromosomal arrays

| **Name** | **Constructs** | **Co-injection marker** | **Figure** |
| --- | --- | --- | --- |
| *wyEx8392* | pOL036, pXD26, pXD379 | P*odr-1::rfp* | 1 |
| *wyEx8151* | pXD331 | P*unc-122::rfp* | 1S1 |
| *wyEx8158* | pXD346 | P*unc-122::rfp* | 2 |
| *wyEx5781* | pXD38, pXD86, pOL036 | P*odr-1::gfp* | 2S3 |
| *wyEx6623* | pXD156, 20ng/μL | P*unc-122::rfp* | 1 |
| *wyEx6624* | pXD156, 0.5ng/μL | P*unc-122::rfp* | 3, 3S1 |
| *wyEx8391* | pXD380 | P*unc-122::rfp* | 3S1 |
| *wyEx7314* | pOL071, pXD69 | P*unc-122:rfp* | 4S2 |
| *wyEx4288* | Fosmid WRM0613aH08 | P*myo-2::mCherry* | 5 |
| *wyEx7806* | pWZ99 | P*myo-2::mCherry*+P*myo-3::mCherry* | 5 |

**Table S2. Plasmids used in this study**

1. Plasmids for worm transgenes

| **Plasmid** | **Description** | **Note** |
| --- | --- | --- |
| pOL036 | *ser2prom3::myr-mCherry* |  |
| pXD26 | P*dpy-7::sax-7s:yfp* |  |
| pXD379 | P*nhr-81::cfp* |  |
| pXD331 | *hsp16.48::kpc-1a* |  |
| pXD346 | *hsp16.48::dma-1* |  |
| pOL020 | *ser2prom3::myr-gfp* |  |
| pWZ54 | *ser2prom3::hpo-30::gfp* |  |
| pWZ55 | *ser2prom3::dma-1::gfp* |  |
| pXD156 | *ser2prom3::kpc-1a* |  |
| pOL071 | P*nhr-81::sax-7s* |  |
| pXD69 | P*nhr-81:mnr-1* |  |
| pXD380 | *ser2prom3::kpc-1ΔPro* | Lack amino acids 37-144 |
| pWZ99 | *ser2prom3::dma-1Δcyto* | Last 68 amino acids deleted |
| pXD38 | P*mec-17::sax-7s::yfp* |  |
| pXD86 | P*mec-17::mnr-1* |  |
| pXD302 | *ser2prom3::dma-1Δcyto::gfp* | Entire cytosolic domain truncated except the first 3 amino acids after TM |

1. Plasmids for *Drosophila* S2 cell transfection

| **Plasmid** | **Description** |
| --- | --- |
| pXD49 | Pa*ctin::sax-7s::gfp* |
| pXD54 | Pa*ctin::dma-1::rfp* |
| pXD55 | P*actin::dma-1::Myc* |
| pXD85 | Pa*ctin::mnr-1::gfp* |
| pXD199 | P*actin::kpc-1::gfp* |
| pXD207 | P*actin::kpc-1::HA* |
| pXD368 | Pa*ctin::kpc-1R136AR143A::HA* |
| pXD242 | Pa*ctin::mCD8::venus* |
| pXD231 | Pa*ctin::kpc-1::cfp* |
| pXD236 | Pa*ctin::venus::rab-7* |

**Table S3. sgRNAs and repair oligos for CRISPR**

| **Strain** |  | **Sequence** |
| --- | --- | --- |
| *dma-1(wy908)* | sgRNA | ttCGTGAGCGACAGTACaa |
| *dma-1(wy1041)* | sgRNA | ttCGTGAGCGACAGTACaa |
|  | Repair oligo | ttcttatgattcttggatgcatctatttccttCGTGAGCGtCAaTAtGGCAGCGGATCCGACTACAAGGACGACGATGACAAGGATTAtAAaGAtGACGAcGAtAAaggcagcggaagtaaGggatcatatgtgacacgagaacactctcgaactccactca |
| *kpc-1(wy1060)* | sgRNA | GGAACAGCAAGTGGCGAAG |
|  | Repair oligo | GGAAGATGTCATGTGGATGGAACAGCAAGTGGCGAAGCGAgcAGTGAAAAGAGGATATCGAgcGATTCGACGACATACTGATGATAATGATATTTTTGAAGAGGATGATGATG |
